# Families in quarantine for COVID-19 in Italy. Resilience as a buffer of parental distress and problematic children’s emotions and behaviors

**DOI:** 10.1007/s12144-022-03374-7

**Published:** 2022-07-25

**Authors:** E. Pugliese, O. Mosca, D. Paolini, F. Mancini, D. Puntonieri, F. Maricchiolo

**Affiliations:** 1 Associazione Scuola di Psicoterapia Cognitiva (APC-SPC), Rome, Italy; 2 Department of Education, Psychology, Philosophy, University of Cagliari, 09123 Cagliari, Italy; 3 Department of Human Science, Italian University Line (IUL), Florence, Italy; 4 Department of Human Sciences, Marconi University, Rome, Italy; 5 ASP Cosenza, Cosenza, Italy; 6 Department of Education, University of Roma Tre, Rome, Italy

**Keywords:** Family, Resilience, COVID-19, Emotional and behavioral problems of children, Distress, Anxiety, Stress

## Abstract

The pandemic of Covid-19 has had a high impact on people’s lives and especially on families. In Italy, in 2020, the several forced closures led families to live indoors to manage anxiety and distress. It was considered appropriate to investigate which protective factors, like parental resilience, can mitigate the negative impact of pandemic-related distress on family life. We have conducted two online surveys during different national lockdowns for Covid-19. The first survey was conducted immediately after the disruption of the virus and the second one after nine months. We measured parental resilience and distress, anxiety, problematic behaviors, and somatization of their children (as assessed by the parents). The aim was to investigate the protective role of parental resilience in mitigating parental distress and in turn problematic emotional states and behavior of their children. Mediation analyses confirmed the hypothesis that parental resilience lowers parental distress and consequently the anxiety and behavioral disorders of their children in both acute distress (first study) and chronic distress (second study) situations. Such results suggest that the improvement of parents’ resilience can buffer the negative impact of pandemic-related parental distress and children’s behavioral problems on both occasions. The need for focused interventions and treatments aimed to reinforce parental resilience is discussed. Targeted prevention and support strategies are needed now, and early in case of future health crises.

## Introduction

In the last two years, the threat of a global pandemic from viral infection has become a dominant international health concern. More than 230 million individuals have been infected all over the world, and almost five million people have lost their lives due to Covid-19, at the time of writing (https://covid19.who.int/). Moreover, in 2020 and 2021, the health systems were collapsing causing widespread social and economic disruption. To face this serious situation and to preserve physical health from the risk of contagion, hospitalization, and death, most governments tried to reduce the contagion. They implemented several drastic security measures such as forced social isolation, quarantine, curfew, etc. (Van Bavel et al., 2020; Zhou et al., 2020). As shown by a wide amount of research, the restrictive home confinement measures dramatically affected the psychological health of people. People experienced a high level of anxiety, fear, and worry, sleep difficulties, depression, panic and stress disorder, psychological trauma, and difficulty in emotional regulation, as well as concerns for personal and close others’ health (Prime et al., 2020; Singh et al., 2020; Cao et al., 2020; Cellini et al., 2020; Kachanoff et al., 2021; World Health Organization, 2020; Qiu et al., 2020; Van Bavel et al., 2020). Moreover, a study by Zalsman et al. (2021) showed that during the first lockdown (April–May 2020) suicide-related calls to a national crisis chat hotline sharply increased (48% compared to the same period in 2019). Notably, this increase exactly corresponded to the total forced lockdown period.

Another study (Orgilés et al., 2020), focused on the psychological effects of Covid-19 quarantine on youth from Italy and Spain (3–18 years old). The study’s results showed that 85.7% of the parents perceived changes in their children’s emotional state and behaviors. The most frequent symptoms were difficulty concentrating (76.6%), boredom (52%), irritability (39%), restlessness (38.8%), nervousness (38%), feelings of loneliness (31.3%), uneasiness (30.4%), and worries (30.1%). Spanish parents reported more symptoms than Italians. Concerning the use of monitors, as expected, parents reported an increased use by children of both countries, less time spent doing physical activity, and hours of sleep during the quarantine. Furthermore, when family coexistence during quarantine became more difficult, the situation was more serious, and the level of distress was higher, parents tended to report more emotional problems in their children.

The scientific literature on previous pandemics and lockdowns’ psychological impacts on the general population, parents, and children, confirmed this negative trend. For example, initial research on SARS in China showed high levels of fear, depression, and emotional distress among the general population in the most highly exposed areas (Yueqin et al., 2003; Qian et al., 2003; Yu et al., 2005) and with greater rates of suicide (Chan et al., 2006). Brooks et al. (2020), examined studies relating to the impact of long-term lockdown on mental health and psychological well-being during the Sars, Mers, and Ebola epidemics. They found that only one study (Sprang & Silman, 2013) was focused on the psychosocial responses of children and their parents to pandemic disasters: 30% of children and 25% of parents in lockdown had posttraumatic stress disorder. Other studies referring only to the adult population revealed the negative influence of isolation and confinement on many psychological symptoms - mood disorders, irritability, insomnia - and physiological changes - dyspnea, arterial hypoxia, headaches, hypocapnia, hyperventilation, suppression of the immune system and hyperthyroidism (Bodey, 1974; Guenter et al., 1970; Muchmore et al., 1974; Reed et al., 1986). These data indicated the need for social-psychological and clinical interventions to support and improve all family members’ well-being. For example, an increase in depression symptoms following long-term exposure to an isolated, confined environment has been shown in existing research (Gunderson, 1963; Kanas, 1987; Strange & Youngman, 1971; Bueno-Notivol et al., 2021).

Because of the Covid-19 crisis, there have been several studies examining the adverse psychological effects (depression, stress, post-traumatic stress disorder, etc.) of state-imposed lockdowns (e.g., Wang et al., 2020; Choi et al., 2020; Solomou & Constantinidou, 2020; Bartoszek et al., 2020; Roma et al., 2020). All these evidences have revealed the often-hidden fragility of families. This latter may have been a source of anxiety, depression, and stress (distress) for parents, and emotional and behavioral discomforts for their children. Moreover, adverse effects in terms of mental and psychosocial health in parents, children, and adolescents in the short term could also be extended in the long-term period (Cluver et al., 2020). To prevent the worsening of psychological symptoms is fundamental to consider individual risks, and so the ones experienced by parents and by children separately, but also the interaction between them which can provide information about different relational levels, e.g., parents-children relationships or couple dynamics (Di Giorgio et al., 2021; Morelli et al., 2020). Notably, recent findings have revealed that parents tend to report greater distress than nonparents during the global Covid-19 pandemic (Park et al., 2020; Russell et al., 2020). Furthermore, motherhood, individual psychological distress, and having younger children can be considered predictive factors of greater parent exhaustion (Marchetti et al., 2020). Although there are several vaccines for the virus Covid-19, the ability, and the effectiveness of those vaccines to prevent infection or disease could be reduced by the new variants (Lopez Bernal et al., 2021). Moreover, different societal factors had exacerbated the negative reactions experienced during the lockdowns: for example, recently, Bagus et al. (2021) demonstrated that mass and digital media communication had adverse consequences during the Covid-19 crisis, leading people to an overestimation of threat (Ioannidis, 2021), resulting in collective hysteria. Concerning Italy, the nation has not yet defeated Covid-19 despite being the European country with the higher number of fully vaccinated people. Italy has been the first European country which implemented the restrictive measures early described and will be the last nation to loosen restrictions. Even if control measures will be loosened soon, we can expect a huge impact of this pandemic in the long-term period. So, an intervention to sustain families is much needed. The regulation and resolution of a pandemic depend on a great extent on government policies and measures, activities of other community members as well as the capacity of health institutions to provide adequate services to individuals promptly. Hence, interventions to reduce the negative long-term impact of the last years of closures and improve the well-being of parents and their children (Orgilés et al., 2020) should be considered fundamental. Furthermore, in agreement with Dohrenwend (1978) and concerning crisis conditions in general, a preventive intervention on possible future crises could moderate the negative effects resulting from this stressful condition. Such an intervention may strengthen psychological buffers from prolonged adverse effects of crises on family well-being.

In line with that, a study conducted during the Covid-19 pandemic in U.S. adults (Killgore et al., 2020) found that psychological resilience helped to face the crisis; moreover, resilience was predicted by psychosocial support and related to modifiable factors, suggesting that it can be promoted and strengthened. In the last two decades, this construct has become a key variable in mental health theory and research. It can be defined as the individual ability to resist, recover and even grow after stress, adversity, crisis, trauma, disease, and disasters (Jakovljevic, 2018), promoting adaptation (Wagnild & Young, 1993). As Walsh (2003) stated “although some families are shattered by crisis or chronic stresses, what is remarkable is that many others emerge strengthened and more resourceful” (cit. p.1). The quarantine as a special stress condition damaged the functions of the whole family system with relevant consequences in terms of mental health in the short and long period (Calvano et al., 2021; Gadermann et al., 2021). Hence, it is primary to deepen the understanding of the impact that the confinement experience would have on the psychological health of children and their families (Fontanesi et al., 2020). Moreover, this condition underlines the importance of finding psychological buffers that can protect families and their children from any future quarantines. Families were the only social system to which children refereed in lockdown (Cobham et al., 2016; Singh et al., 2020); it is, therefore, important to pay attention to good parenting skills such as facing adverse situations, resisting distress, and promoting a positive adaptation to temporary life changes and family well-being. These aspects were not sufficiently analyzed in the scientific literature (Fontanesi et al., 2020), especially concerning lockdown-specific processes.

A study conducted in Italy by Spinelli et al. (2020) during the first lock-down showed that the perception of forced home-confinement impacted children’s behavioral and emotional problems through the influence, i.e. mediation, of parent’s individual and dyadic distress, with a stronger effect from the latter.

## The Present Study

Analyzing parents’ and children’s reactions and emotions, and identifying risk and protective factors is essential to properly address their needs and tailor intervention programs (Sprang & Silman, 2013). Moreover, according to the Family Stress Model, parents’ subjective perceptions of financial/psychosocial stress (both acute and chronic stress) exacerbate their negative feelings such as worry and sadness, which could lead to parents and children’s psychological and relational problems (Conger et al., 2000; Masarik & Conger, 2017). We consider the Family Stress Model (FSM) as a useful framework for understanding the family stress process and its potential effect on children’s mental health and for the first time in the particular environmental condition of the pandemia for Covid-19.

So, based on these considerations, FSM and research on family stress, coping, and adaptation (Hill, 1958; McCubbin & Patterson, 1983; Patterson, 1988, 2002; Conger et al., 2000; Masarik & Conger, 2017), the present research adopted a cross-sectional design methodology and was conducted in two different periods of lockdown with different samples of participants: the first study (Study 1) was launched in March 2020, full lockdown period (the data were collected in April 2020 and analyzed in May and June 2020); while the other data collection (Study 2) was launched during the second national lockdown, nine months after the first forced isolation (data were collected during December 2020). One of the aims of the first study was to investigate the immediate psychological effects of unpredictable social isolation and understand how parents dealt, in terms of resilience, with the emotional distress associated with this unprecedented and global emergency (i.e. acute stress condition and global trauma). So different than the study conducted by Spinelli et al. (2020), we focused on the mediational role of distress in the relationship between a protective factor like resilience and children’s difficulties in managing emotional and behavioral issues. The second aim of Study 1 was to understand how this severe distress influenced the perceptions of children’s problematic emotions and behaviors as evaluated by the parents. Particularly, we tested the mediational role of parental distress (i.e., depression, anxiety, and stress level) on the relationship between parent resilience and both anxiety and problematic behaviors of their children (Study 1). We expected that the implications of the Covid-19 outbreak might increase parents’ distress with a consequent negative impact on children’s emotional and behavioral well-being (Dalton et al., 2020).

- H1: Resilience may reduce parental distress
- H2: Resilience may reduce children’s problematic behaviors and anxiety
- (direct effect)
- H3: Resilience may reduce children’s problematic behaviors and anxiety through the reduction of parental distress (indirect effect)

In Study 2 we have tested the same hypotheses but in relation to a chronic stress condition. In fact, at the time of data collection (December 2020, after nine months of the pandemic) it was possible to distinguish between acute and chronic stress conditions. Moreover, it is well known and understandable that COVID-19 results in greater adverse outcomes and a higher risk for mortality in patients with pre-existing chronic medical conditions compared to healthy patients (Gabrielli & Lund, 2020). The same principle could be applied to social and psychological demands related to the societal impact of Covid-19. To understand the family distress, it is important to highlight that stress can be acute or chronic during the different lockdown research periods. Referring to the DSM-5, “Acute Stress Reaction refers to the development of transient emotional, cognitive, and behavioral symptoms in response to an exceptional stressor such as an overwhelming traumatic experience involving serious threats to the security or physical integrity of the individual or of a loved person(s) (e.g., natural catastrophe, accident, battle, criminal assault, rape), or an unusually sudden and threatening change in the social position and/or network of the individual, such as the loss of one’s family in a natural disaster” (American Psychiatric Association, 2013). On the other hand, chronic stress is defined in the APA dictionary as “the physiological or psychological response to a prolonged internal or external stressful event (i.e., a stressor). The stressor need not remain physically present to have its effects; recollections of it can substitute for its presence and sustain chronic stress” (American Psychiatric Association, 2013). Thus, acute stress differs from the concept of chronic stress which is based on the intensity, frequency, and duration of stressors (Gannon & Pardie, 1989). We have used the distinction of acute and chronic stress in relation to the different main times in the pandemic for Covid-19 at the time of writing: the first forced quarantine (March 2020), can be considered as a global trauma that corresponds to the definition of acute stress (American Psychiatric Association, 2013); in the second forced lock-down (December 2020), the pandemic was still in its peak and no permanent solution was settled at that moment and this condition of prolonged experienced difficulties with no apparent possibility to change the situation corresponds to the definition of chronic stress (American Psychiatric Association, 2013). So, in our framework, the distress is the mediator variable and the COVID-19 pandemic and lockdowns are the stressors.

## Study 1

### Methods

#### Participants

We recruited 384 Italian participants by spreading an online survey. We selected participants who declared to have a child without disability and a psychiatrist diagnosis. The remaining sample was composed of 292 participants (mean age = 42.73, SD = 6.08) of which mothers *n* = 253 (87%) and fathers *n* = 39 (13%). The majority of the sample (84.2%) was composed of married/cohabiting couples, 12.3% of divorced/separated, 2.4% of singles, and 1% of widows/widowers. Concerning the educational background of the sample: 0.3% had no title, 0.3% had a primary license, 44.9% held a high school diploma, 16.8% had a bachelor’s degree, and 37.7.2% had a post-laureate title. 8.3% (n = 14) of the sample lost their job due to the Covid-19 pandemic. Participants took part in the survey voluntarily.

#### Procedure

The questionnaire was implemented by using the Google form platform. We did not ask participants to disclose personal data and we have ensured the right of anonymity for all respondents. The survey was live from the 4th of April to the 3rd of May 2020 (the end of forced quarantine). Participants were recruited by snowball sampling, posting the survey link on social networks, and directly inviting patients treated by different private psychotherapists.

The questionnaire took approximately 30 minutes to fill in. According to the ethical standards Declaration of Helsinki (World Medical Association, 2001), participants were informed about all relevant aspects of the study (e.g., methods, institutional affiliations of the researchers) before they started to fill out the questionnaire. Importantly, they were apprised of their right to anonymity, to refuse and to participate in the study, or to withdraw their consent at any time during the study without fear of reprisal. Participants then confirmed that they had understood the instructions correctly and agreed to participate. The research protocol was approved by the local Ethics Committee of the School of Cognitive Psychotherapy (Scuola di Psicoterapia Cognitiva Srl, N Pr. 2/20).

#### Materials

##### Resilience

Participants’ level of Resilience was assessed by using the Italian version of the Resilience Scale (Callegari et al., 2016). Participants filled out 14 items (e.g., “*I am a friend of myself*”; “*Usually I find something to smile about*”) ranging on a Likert-type scale from 1 (Completely Disagree) to 7 (Completely Agree) thinking on how each sentence describe you during the lockdown. We averaged responses – after reverse-coding negative items – to create an overall parental resilience index (α = .90), in which higher ratings indicated higher parental resilience.

##### Distress

To evaluate the participants’ level of general distress, we used the *Depression Anxiety Stress Scales-21* (DASS; Bottesi et al., 2015). It includes 21 items that are grouped into 3 subscales assessing people’s level of Depression (7 items; “I felt discouraged and depressed”); Anxiety (7 items; “I realized that my mouth was dry”) and Stress (7 items; “I have tended to overreact to the situations”). To capture pandemic-related distress, we adapted the Scale explicitly asking participants to fill it out by thinking of how they felt during the COVID-19 lockdown on a Likert-type scale from 0 (It never happened to me to 3 (It almost always happened to me). To create a single index for general distress (α = .96), we averaged responses for the three subscales scores. Higher ratings indicated a higher level of distress.

##### Children’s Anxiety and Problematic Behaviors

Participants were asked to fill two subscales of the Child & Adolescent Behavior Inventory scale (C.A.B.I., Child & Adolescent Behavior Inventory, Cianchetti et al., 2017). The first one is composed of 25 items and is addressed to evaluate the level of children’s anxiety (i.e., “*He/she looks tense and/or anxious*”; “*He/she worries too much about the school*”; *α =* .88). While the second one included 19 items and was focused to evaluate the level of children’s problematic behaviors (i.e., “*He/she does not respect the rules*”; “*He/she destroys objects*”; α = .88). Participants could answer each item by choosing from three alternative responses (i.e., True, Sometimes true, False). For each subscale, we first summed the item per participant, and then we averaged responses to create two different indexes. Higher ratings indicated a higher level of children’s anxiety and a higher level of children’s problematic behaviors respectively.

## Results

Table 1 shows the means and standard deviations among all variables and the correlations between all measures investigated in the study. All variables are significantly related to each other.

**Table 1:** Means (standard deviation), and zero-order correlations among variables (*n* = 292)

	Mean (SD)	1	2	3	4
1. Parental resilience	5.80 (.86)	1			
2. Parental distress	0.78 (.67)	−.499*	1		
3. Children anxiety	9.92 (7.59)	−.329*	.478*	1	
4. Children problematic behaviors	6.05 (5.85)	−.274*	.347*	.506*	1

*p < .05

### Mediation Analyses

To test our hypotheses, we conducted two mediation analyses (PROCESS model 4) by using the SPSS macro developed by Hayes and Preacher (2014), in which parents’ resilience was inserted as an independent variable, parents’ distress as a mediator, and the level of children’s anxiety and problematic behaviors as dependent variables, separately.^1^ We have added gender and age as covariates of the model but both variables were not significant.

**Fig. 1:**
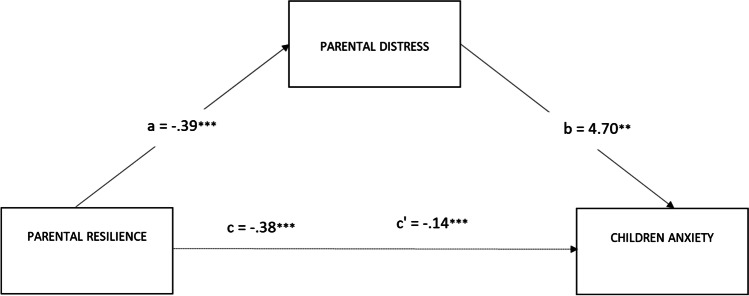
Parental distress mediates the effect of parental resilience on the level of children’s anxiety. Notes: ***p < 0.001; **p < 0.01; *p < 0.05

The first model in which the relationship between the parents’ resilience and the children’s anxiety was mediated by the parents’ distress was significant: *R*^*2*^ = 0.24; F (2, 289) = 45.43, *p* < 0.001 (see Fig. 1). The bootstrap analysis with 5000 resampling showed that the indirect effect of parents’ resilience on the level of children’s anxiety through parents’ distress level was significant (*b* = −1.83; 95% CI: LLCI = −2.5348; ULCI = −1.2576), as well as the direct effect between parents’ resilience and the level of children’s anxiety (*b* = −1.07; 95% CI: LLCI = −2.0909; ULCI = − 0.0425).

**Fig. 2:**
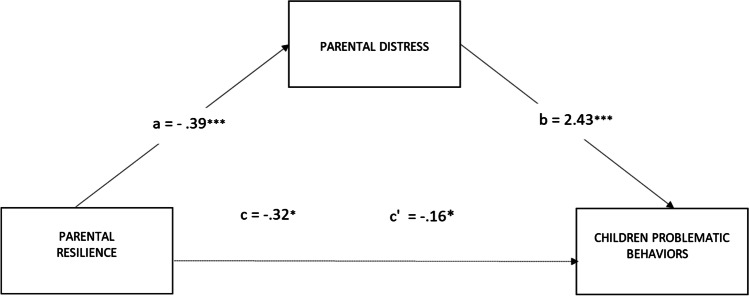
Parental distress mediates the effect of parental resilience on the level of children problematic behaviors. Notes: ***p < 0.001; **p < 0.01; *p < 0.05

The second model in which the relationship between parents’ resilience and children’s problematic behaviors was mediated by the parents’ distress was significant: *R*^*2*^ = 0.13; F (2, 289) = 22.35, *p* < 0.001 (see Fig. 2). The bootstrap analysis showed that the indirect effect of parents’ resilience on the level of children’s problematic behaviors through parents’ distress level was significant (*b* = −0.95; 95% CI: LLCI = −1.5125; ULCI = −0.4934), as well as the direct effect between parents’ resilience and the level of children’s problematic behaviors (*b* = −0.91; 95% CI: LLCI = −1.7504; ULCI = −0.0657).

Overall, results indicate that the increase in parents’ resilience predicts a decrease in the level of both children’s anxiety and problematic behaviors during the lockdown. This relationship is explained by a decrease in the level of parents’ distress.

## Study 2

### Participants

We recruited 168 Italian participants by spreading an online survey (mean age = 42.73 SD = 6.07) of which mothers *n* = (89%) and fathers *n* = (11%). The majority of the sample (81%) was composed of married/cohabiting couples, 9.5% divorced/separated, 7.7% of singles, 0.6% widows/widowers, and 1.2% single mothers. Concerning the educational background of the sample: 35.1% held a high school diploma, 34.5% a bachelor’s degree, 27.4% a master’s degree, and 23.2% a post-laureate title, 8.3% (n = 14) of the sample lost their job due to the Covid-19 pandemic. Participants voluntarily took part in the survey.

### Procedure

The questionnaire was implemented by using the Google form platform. We did not ask participants to disclose personal data, so we ensured the right of anonymity for all respondents. The survey was live during the Christmas lock-down (i.e., alternating periods of full lock-down, especially during the Christmas holidays, with days with limited mobility; December 2020). Participants were recruited by snowball sampling by posting the survey link on social networks.

Other details (number of protocol and informed consent) were the same of Study 1.

### Materials

In the second study, we administered the same measures as Study 1. Like in Study 1, we adapted the scales explicitly asking participants to fill them out by thinking of how they felt during the second COVID-19 Christmas lockdown, so the measures were pandemic-related.

Reliability of the same measures used in Study 1 are the following: Resilience Scale: α = .88; Distress Scale: α = .95; Anxiety in Children: α = .90; Problematic Behaviors: α = .92; Somatization = α = .70.

## Results

Table 2 shows the means and standard deviations among all variables and the correlations between all measures investigated in the study.

**Table 2:** Means (standard deviation), and zero-order correlations among variables (*n* = 165)

	Mean(SD)	1	2	3	4	5	6
Parental Resilience	5.85(.71)	1					
Parental Distress	0.96(.70)	−.38^**^	1				
Children Anxiety	9.91(8.54)	−.29**	.41**	1			
Children Prolematic Behaviors	5.4(6.08)	−0.14	.38**	.54**	1		
Children Somatization	0.49(.46)	−.22**	.25**	.69**	.39**	1	

** *p* < .01. * *p* < .05

### Mediation Analyses

Like in Study 1, to test our hypotheses, we conducted three mediation models (PROCESS model 4) by using the SPSS macro developed by Hayes and Preacher (2014), in which parents’ resilience was inserted as an independent variable, the parents’ distress as a mediator, and the level of children’s anxiety, somatization and problematic behaviors as dependent variables. Coherently with Study 1, we have added gender and age as covariates of the model but both variables were not significant.

**Fig. 3:**
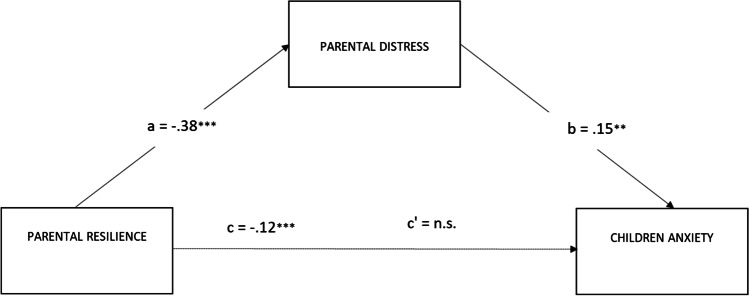
Parental distress mediates the effect of parental resilience on the level of children’s anxiety. Notes: ***p < 0.001; **p < 0.01; *p < 0.05

The first model in which the relationship between parents’ resilience and children anxiety was mediated by parents’ distress was significant: *R*^*2*^ = 0.19; F (2, 162) = 14.26 *p* < 0.001 (see Fig. 3). The bootstrap analysis showed that the indirect effect of parents’ resilience on children’s anxiety through parents’ distress level was significant (*b* = −.0580; 95% CI: LLCI = −.1129; ULCI = −0.0209); however, the direct effect between parents’ resilience and children’s anxiety was not significant (*b* = −0.660; 95% CI: LLCI = −.1437; ULCI = −.0116).

**Fig. 4:**
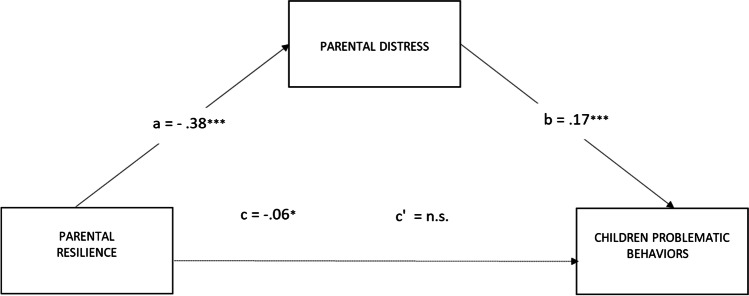
Parental distress mediates the effect of parental resilience on the level of children’s problematic behaviors. Notes: ***p < 0.001; **p < 0.01; *p < 0.05

The second model in which the relationship between parents’ resilience and children’s problematic behaviors was mediated by parents’ distress was significant: *R*^*2*^ = 0.15; F (2, 162) = 8.52, *p* < 0.001 (see Fig. 4). The bootstrap analysis showed that the indirect effect of parents’ resilience on the level of children’s problematic behaviors through parents’ distress level was significant (*b* = −.0666; 95% CI: LLCI = −.1308; ULCI = −.0219); however, the direct effect between parents’ resilience and the level of children’s problematic behaviors was not significant (*b* = .0111; 95% CI: LLCI = −.0697; ULCI = .0719).

**Fig. 5:**
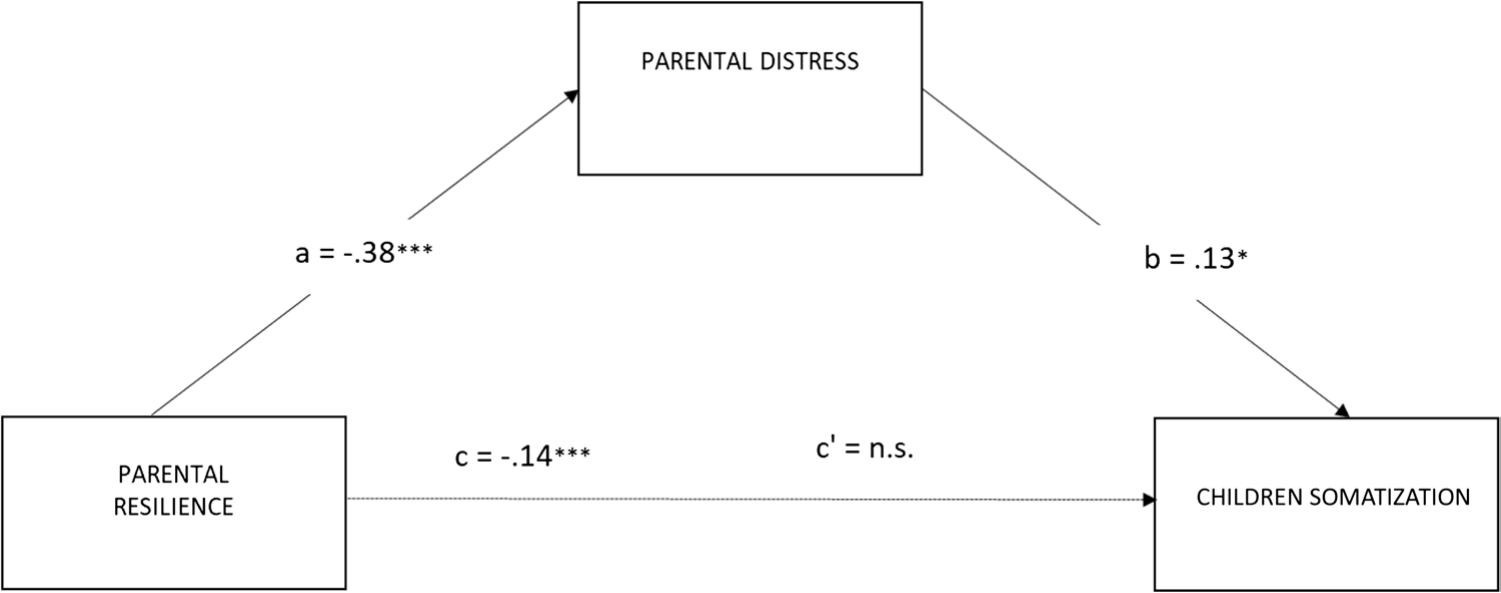
Parental distress mediates the effect of parental resilience on the level of children’s somatization

The third model in which the relationship between parents’ resilience and children’s somatization was mediated by parents’ distress was significant: *R*^*2*^ = 0.15; F (2, 162) = 36.12, *p* < 0.001 (see Fig. 5). The bootstrap analysis showed that the indirect effect of parents’ resilience on the level of children’s problematic behaviors through parents’ distress level was significant (*b* = − .0494; 95% CI: LLCI = −.1132; ULCI = −.0053); however, the direct effect between parents’ resilience and the level of children’s problematic behaviors was not significant (*b* = −.0949; 95% CI: LLCI = −.1999; ULCI = −.0102).

Overall, results indicate that the increase in parents’ resilience predicts a decrease in the level of both children’s anxiety and problematic behaviors during the lockdown. This relationship is explained by a decrease in the level of parents’ distress.

## Discussion

The long restrictive lockdown imposed by the Italian Government forced millions of families (in Italy there are about 26 million families, ISTAT data, 2019) to remain indoors, in close contact, facing an uncertain and anxious period. We have been exposed for more than two years to ongoing threats to our lives and our loved ones, over which we had no control. Many of us have been worried not only for our health but also for our economic future. The negative impact of the COVID-19 pandemic on people’s lives and their mental health, as well as on communities’ social and economic life has been considerable (Cao et al., 2020; Cellini et al., 2020; Kachanoff et al., 2021; World Health Organization, 2020; Qiu et al., 2020; Van Bavel et al., 2020; Paolini et al., 2020). At the moment, we are not yet able to quantify the damage that this stressful period will have in our near future. We can only try to understand how to protect ourselves from the inevitable negative consequences of this period. Based on the Family Stress Model (FSM), our explorative study aimed to respond to this imperative. We investigated the impact of home confinement for Covid-19 on the psychological well-being of parents and their children. Specifically, we considered the protective role of adult resilience on distress and in turn on dysfunctional behaviors of children as observed by the parents. Results showed that parents’ resilience and distress, and children’s anxiety and behavioral problems were significantly intercorrelated. Prime et al. (2020) depicted a process through which the COVID-19 pandemic affected children, caregivers, and the whole family system. The heavy and traumatic social disruption (e.g. forced quarantine, unexpected job loss, traumatic human losses, etc.) brought by COVID-19 detrimentally influenced children and parents’ well-being and these effects may also be bidirectional (Eales et al., 2021). In the framework of FSM, a change in one family member’s functioning can affect the functioning of the entire family system (Conger et al., 2000; Masarik & Conger, 2017; see also Eales et al., 2021).

Our analysis confirmed this framework: the increase in parents’ resilience significantly predicted a decrease in the level of children’s anxiety and behavioral symptoms. This relationship was explained by a decrease (mediation effect) in the level of parents’ distress both following an acute (Study 1) and a chronic stress (Study 2).

Resilience has been a protective factor and a resource for the mental health and well-being of the whole family (Moscardino et al., 2021; Sorkkila & Aunola, 2021). Our analysis inspired an interesting observation: the mediation of parental distress in Study 1 is partial and in Study 2 total. This result can be interpreted because of the amount of time families were exposed to stress due to the pandemic, or chronic stress (Maslach, 2003). In Italy, at the time of writing, even if the state of emergency was going to be ended the Government has decided to strengthen the restrictive measures in extending the Green Pass, i.e., the EU Digital COVID Certificate Regulation which entered into application on 01 July 2021, to all the workers to maintain their job.

If in March–May 2020 (Study 1) the first lock-down represented a sudden break for families (a condition of acute stress), the second lock-down during Christmas 2020 (Study 2) seemed to be more a period of chronic exposure to the stress of nine months of pandemic (a condition more assimilated to chronic stress). This would also explain the total meditation’s effect of parental distress on children’s somatization (which was not significant in the first study). Somatization, in fact, among the adverse effects of traumatic events in relation to acute stress, appears more in the long term than anxiety and behavioral dysregulation, which are generally more immediate reactions. It is only possible to hypothesize that Italian families were at that time more in a condition of chronic stress. This kind of stress may be assimilable to the burn-out recorded for health workers (Di Trani et al., 2021) or to the parental burnout in the Portuguese families (Aguiar et al., 2021) during this pandemic. Specifically, the parental burnout has been defined as a stress-related syndrome that consists of emotional exhaustion as a parent (i.e., chronic fatigue that does not go away by resting), being fed up as a parent (i.e., not enjoying parenting anymore), emotional distancing from children (i.e., parent can perform only the instrumental aspects of parenting but the warmth disappears), and contrast in previous parental self (i.e., parents feel no longer as good parents as they once were). This is often accompanied by feelings of guilt and shame (Roskam et al., 2018). Parental burnout can be a result of exposure to chronic parenting-related stressors, where the demands constantly exceed the parents’ resources (Mikolajczak & Roskam, 2018). The demands are stress-producing factors (e.g., forced smart-working, housework overload, high parental demands, lock-downs) and the resources are stress-alleviating factors (e.g., resilience, emotional support, self-compassion as a parent) (Sorkkila & Aunola, 2021).

Our study has evident limitations linked to its correlational character and the limited sample; it would be advisable in future studies to increase the number of families involved, also considering additional factors, e.g. presence of disability or diseases in the family, difficult economic conditions, and especially couple conflicts, as well as to investigate their impact on family distress during this pandemic period (Smith et al., 2020). The role of resilience in reducing such impact needs to be investigated specifically. Longitudinal studies are recommended to highlight with certainty the effectiveness and the influence of parents’ resilience on their anxiety and the emotions and behaviors of their children, during the emergency periods. In both studies, we did not measure the level of anxiety and problematic behaviors in children before the pandemic. So, we did not control for these factors treating them as covariates, allowing us to detect floor or roof effects. Indeed, we adopted the strategy to exclude from our dataset children with disabilities and diagnoses to partially address this limitation. The same considerations could be applied to parental distress, at least for Study 1, in which the sample was recruited also directly inviting patients treated by different private psychotherapists. For this reason, we can’t exclude that respondents were already stressed before the pandemic disruption. In Study 2 we have partially addressed these limitations in adopting a snowball procedure sampling. Another limitation of our study is that children’s anxiety and dysfunctional behaviors were assessed by only one parent which responded to the questionnaire and not by both parents or by children themselves (as self-reported measures or systematic observation by researchers). Future studies should take into consideration this aspect, measuring such variables through the assessment of both parents and/or by the direct observation of the children. This last method was impossible for us during the quarantine period of our data collection because of the restrictions. Moreover, future studies could investigate which social factors may predict the psychological reactions of Italian families during the Covid-19 restrictive phase, such as family resilience (i.e., group-based resiliency, Pagliaro et al., 2013). Another interesting issue for future studies could be the focus on resilience sub-factors like self-reliance, equanimity, or authenticity (see Callegari et al., 2016). Finally, despite our large efforts spent in recruiting both mothers and fathers, the absolute majority of mothers in our sample, in both studies, limit our results, particularly to them. Some studies which involved specifically fathers (Trumello et al., 2021) helped to disentangle possible differences between motherhood and fatherhood during emergency periods. This line of research should be carried forward.

Despite these limitations, this study is important as it takes a picture of the situation of Italian families during a crisis dragging on over time due to the Covid-19 pandemic not only for health but also in social and psychological terms due to lock-down and forced isolation. It is important to know the protective factors for a healthier family life, which protect against possible future psycho-pathogenic crises, especially in children.

## Implications and Application

The severe social crisis caused by the current pandemic brought us to reflect on the importance of endowing the future society with resilience to face stressful situations functionally and healthily. Such as a family resilience perspective focusing on parental as well as children strengths and resources considers the impact of serious crises and persistent adversity on the whole family.

The study also suggests that resilience, as a protective factor in times of global social crisis, should also be strengthened in children from infancy to provide them with a shield that defends them from pathological anxiety states or dysfunctional behaviors, through targeted educational projects, conducted possibly in schools. Interventions aimed to promote resilience could be a successful strategy for mitigating the negative impact of the pandemic on families.

Therefore, the results of Study 1, confirmed in Study 2, suggested that it is essential to plan interventions aimed at promoting the reinforcement of parents’ resilience. It is also fundamental to encourage resilient reactions to traumatic events in children, as well as to prevent risk situations. These latter aggravate the stress evoked by confinement situations, such as conflicts among a couple or family members. A determinant factor in children’s emotional and behavioral management is parental resilience. Resilience is a dynamic, multi-level, multi-systemic process of positive adaptation (Basu et al., 2022) and for these reasons is full in potential. It can be nurtured and strengthened through targeted clinical and social interventions (Basu et al., 2022). Study 1 slso showed the importance of reducing parental distress. A recent study (Achterberg et al., 2021) showed that perceived stress was a significant mediator for changes in parental negative feelings and children’s externalizing behaviors. These results were confirmed also in a study conducted in Singapore (Chung et al., 2020): levels of parental stress mediated the impact of COVID-19 on harsh parenting and parent-child relationship closeness. Results of Study 2 showed that there is a difference between acute and chronic distress. Indeed, experiencing chronic stress contributes to the development of psychological and emotional difficulties, such as psychosomatic disorders, anxiety, depression, and burnout, which affect functioning at work and in the personal sphere (Maslach, 2003). Since the pandemic is still not outdated and for this reason, many families are probably experiencing high levels of burnout, government efforts to mitigate the economic impact of the pandemic are urgently needed to help financially-distressed families (e.g., financing support, tax, and other temporary relief measures).

Finally, this explorative study would provide indications on intervention and education projects, suggesting a direction on how to face the emerging psychological and social needs for an immediate future. We think that a multidisciplinary intervention approach would be needed including clinical, educational, and social service perspectives aimed to a) support parents on resilience and anxiety regulation to promote family well-being, b) support children’s well-being at school through programs for resilience education and c) improve the identification of family problems through mediation interventions for the management and resolution of conflicts in emergencies.

## Data Availability

The raw data supporting the conclusions of this article will be made available by the authors upon request.

## Footnotes

1 It is worth noting that we did not report non-significant models and that, given the cross-sectional nature of our study, we were not sure that parents weren’t having high levels of distress because their children were misbehaving because they were confined to the home. For this reason, we have conducted mediation analyses also with children’s problematic behaviors and anxiety as mediators and parental distress as outcome. However, explained variances of the models were inferior to the ones reported.
